# How PERK kinase conveys stress signals to nuclear factor-κB to mediate estrogen-induced apoptosis in breast cancer cells?

**DOI:** 10.1038/s41419-018-0516-y

**Published:** 2018-08-13

**Authors:** Ping Fan, V. Craig Jordan

**Affiliations:** Department of Breast Medical Oncology, The University of Texas MD Anderson Cancer Center, Houston, TX USA

Antihormone therapies using a selective estrogen receptor modulator or an aromatase inhibitor are standard strategies for treatment and prevention of estrogen receptor-positive breast cancer^1^. However, acquired resistance to antihormone therapies is inevitable. Paradoxically, estrogen (E_2_)-induced apoptosis was an unanticipated discovery in studies of long-term antihormone therapies that produced selective pressure on breast cancer cells to create selective cell populations vulnerable to E_2_-induced apoptosis in vivo^2^ and in vitro^3,4^. This finding is clinically relevant to low-dose E_2_ treatment of aromatase inhibitor-resistant breast cancer, with a 30% clinical benefit rate^5^, and it is a mechanistic interpretation for the decrease in breast cancer incidence and mortality in the conjugated equine estrogen alone trial performed by the Women’s Health Initiative^6^. All of these clinically relevant findings encouraged us to identify the key molecules involved in E_2_-induced apoptosis to improve the therapeutic effects of E_2_ on endocrine-resistant breast cancer.

Accumulation of stress responses, including endoplasmic reticulum, oxidative, and inflammatory stresses, is a major mechanism of E_2_-induced apoptosis in long-term E_2_-deprived (LTED) breast cancer cells^7,8^. Two cellular organelles—mitochondria and endoplasmic reticulum—participate in mediation of E_2_-induced stresses^8^. Mitochondrial dysfunction leads to the release of reactive oxygen species and impairs redox homeostasis^8^. Three sensors of unfolded protein response—protein kinase RNA-like endoplasmic reticulum kinase (PERK), inositol-requiring protein 1 alpha (IRE1α), and activating transcription factor 6 (ATF-6)—are initially activated by E_2_, each having different functions in the endoplasmic reticulum stress^8,9^. PERK attenuates protein translation, which is identified as an important mediator of E_2_-induced apoptosis^8^, whereas ATF-6 and IRE1α are involved in endoplasmic reticulum-associated degradation of phospholipids^9^. The endoplasmic reticulum stress occurs prior to the oxidative stress after exposure to E_2_ in LTED breast cancer cells^8^. Of note, inhibition of PERK kinase activity completely blocks oxidative stress^10^, indicating close crosstalk between these two stresses. Furthermore, a variety of inflammatory factors, such as interleukin-6, fatty-acid desaturase 1, and tumor necrosis factor alpha (TNFα), are activated by E_2_ with different dynamics^7,8^. Induction of TNFα expression peaks after 3 days of E_2_ treatment and is confirmed to be an important factor that induces apoptosis in LTED MCF-7:5 C cells^8,10^. Nevertheless, how E_2_ induces TNFα is unknown and requires elucidation.

TNFα is well known to be a nuclear factor-κB (NF-κB)-dependent gene^10^. NF-κB is a critical stress-responsive transcription factor. Activated sensors of endoplasmic reticulum stress and associated inflammatory responses can activate NF-κB to modulate stress responses^11^. However, literature contains no reports of activation of NF-κB by E_2_ to mediate stress-associated apoptosis in LTED breast cancer cells. This knowledge is very important to understand E_2_ therapy for aromatase inhibitor-resistant breast cancer. Our recent study demonstrated that E_2_ differentially modulates NF-κB activity depending on the treatment time^10^. E_2_ initially has significant potential to suppress NF-κB activation; in other words, E_2_ completely blocks TNFα-induced activation of NF-κB. The lipid metabolism-associated transcription factor CCAAT/enhancer-binding protein beta (C/EBPβ) is activated by E_2_, which is responsible for suppression of NF-κB activity in LTED MCF-7:5 C cells^10^. This result supports the existence of a trans-repressive relationship between ERα and NF-κB^12^. However, NF-κB p65 DNA-binding activity is increased when E_2_ treatment time is prolonged, leading to the induction of TNFα expression in LTED MCF-7:5 C cells^10^. Unlike the regulatory mechanism of TNFα, activation of NF-κB by E_2_ is independent of the canonical IκBα signaling pathway^10^, suggesting novel modulation of NF-κB directly in the nucleus. This delayed activation of NF-κB by E_2_ also indicates that some other factors are involved in overcoming the initial suppression of NF-κB activity by ERα.

**Fig. 1:**
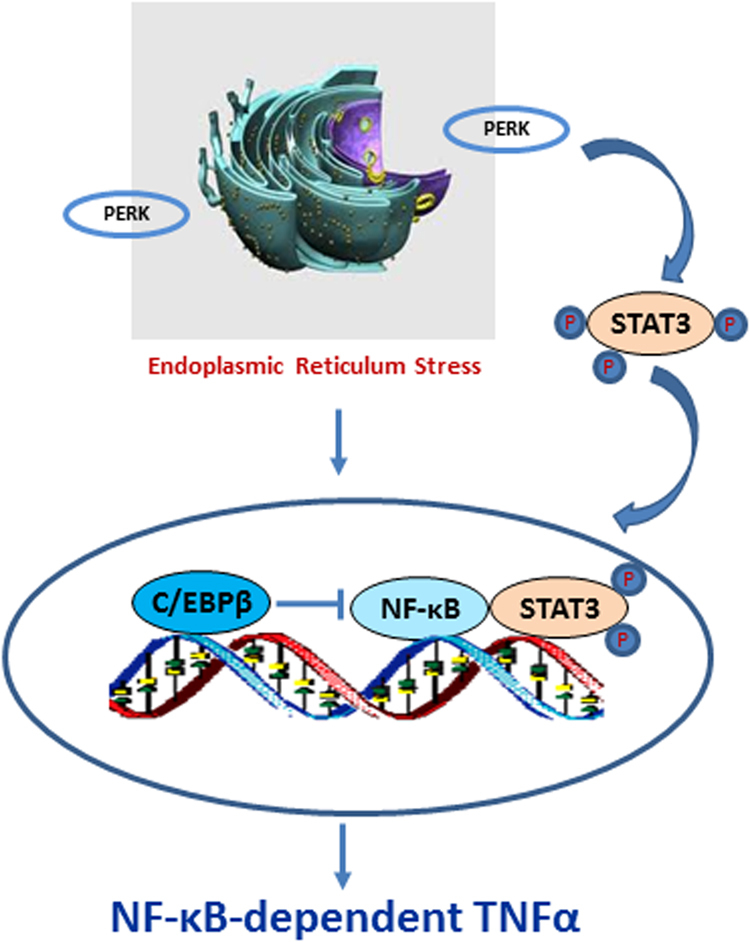
PERK is a key driver that activates the NF-κB/TNFα axis in LTED MCF-7:5 C cells. E_2_ preferentially increases C/EBPβ expression, which suppresses NF-κB DNA binding. E_2_ also activates PERK in response to accumulation of unfolded proteins in the endoplasmic reticulum. This stress kinase phosphorylates STAT3 to increase NF-κB DNA-binding activity, leading to induction of TNFα expression

PERK is a key driver responsible for activation of NF-κB after E_2_ treatment^10^. This is a novel finding that further identifies mechanisms of E_2_-induced apoptosis in LTED breast cancer cells. The basic biological function of PERK is to reduce unfolded proteins in the endoplasmic reticulum by phosphorylating the downstream signal eukaryotic translation initiation factor 2 alpha (eIF2α)^13^. Although eIF2α was reported to activate NF-κB by decreasing the levels of IκBα in mouse embryonic fibroblasts^14^, our results demonstrated that PERK kinase does not rely on eIF2α phosphorylation to activate NF-κB in LTED breast cancer cells^10^. Thus, how does this kinase in the endoplasmic reticulum activate nuclear NF-κB under LTED conditions? Signal transducer and activator of transcription 3 (STAT3) is identified as a stress-responsive mediator that is phosphorylated by PERK to increase NF-κB DNA-binding activity^10^. Furthermore, a specific STAT3 nuclear translocation inhibitor remarkably decreases NF-κB DNA-binding activity, suggesting that a DNA level interaction is sufficient for STAT3 to activate NF-κB. These findings suggest that PERK kinase conveys stress signals from the endoplasmic reticulum to the nucleus through activation of STAT3 and NF-κB under LTED conditions (Fig. 1).

In summary, several stress-responsive transcription factors, including C/EBPβ, NF-κB, and STAT3, participate in stress responses to modulate E_2_-induced apoptosis in LTED breast cancer cells. The regulatory relationship between C/EBPβ and NF-κB also suggests that E_2_-induced apoptosis is closely associated with lipid metabolism. However, the crosstalk between stress responses and transcription factors is complex, depending on the cellular context and inflammatory microenvironment. Despite the fact that MCF-7:5 C and MCF-7:2 A cells are derived from the same parental MCF-7 cells under LTED conditions, NF-κB is constitutively activated in MCF-7:5 C cells but not in MCF-7:2 A cells^10^, whereas MCF-7:2 A cells have a stronger antioxidant system than do MCF-7:5 C cells^15^. These different phenotypes lead to distinctive responses of MCF-7:5 C and MCF-7:2 A cells to E_2_ exposure. For instance, the NF-κB/TNFα axis is highly active in MCF-7:5 C cells but not in MCF-7:2 A cells^10^. Although E_2_ activates PERK similarly in MCF-7:2 A and MCF-7:5 C cells^8,15^, the NF-κB/TNFα axis is not quickly activated in MCF-7:2 A cells in the same way as in MCF-7:5 C cells^10^. This suggests that PERK kinase alone is not sufficient to activate NF-κB, depending on its interactions with other transcription factors. Furthermore, NF-κB can function as a key mediator of oxidative stress^11^. Ongoing studies are focused on how NF-κB modulates oxidative stress in LTED breast cancer cells. These data will provide an important rationale for finding target molecules to improve the therapeutic effects of E_2_-induced apoptosis on endocrine-resistant breast cancer.
